# *In vitro *activity of tigecycline and comparators against carbapenem-susceptible and resistant *Acinetobacter baumannii *clinical isolates in Italy

**DOI:** 10.1186/1476-0711-7-4

**Published:** 2008-02-08

**Authors:** Maria Lina Mezzatesta, Giusi Trovato, Floriana Gona, Vito Mar Nicolosi, Daria Nicolosi, Alessandra Carattoli, Giovanni Fadda, Giuseppe Nicoletti, Stefania Stefani

**Affiliations:** 1 Department of Microbiological and Gynaecological Sciences, University of Catania, Italy; 2 Istituto Superiore di Sanità, Roma, Italy; 3 Department of Microbiology – Policlinico Gemelli Roma, Italy

## Abstract

**Background:**

In a recent multi-centre Italian survey (2003–2004), conducted in 45 laboratories throughout Italy with the aim of monitoring microorganisms responsible for severe infections and their antibiotic resistance, *Acinetobacter baumannii *was isolated from various wards of 9 hospitals as one of the most frequent pathogens. One hundred and seven clinically significant strains of *A. baumannii *isolates were included in this study to determine the *in vitro *activity of tigecycline and comparator agents.

**Methods:**

Tests for the susceptibility to antibiotics were performed by the broth microdilution method as recommended by CLSI guidelines. The following antibiotics were tested: aztreonam, piperacillin/tazobactam, ampicillin/sulbactam, ceftazidime, cefepime, imipenem, meropenem tetracycline, doxycycline, tigecycline, gentamicin, amikacin, ciprofloxacin, colistin, and trimethoprim/sulphametoxazole. The PCR assay was used to determine the presence of OXA, VIM, or IMP genes in the carbapenem resistant strains.

**Results:**

*A. baumannii *showed widespread resistance to ceftazidime, ciprofloxacin and aztreonam in more than 90% of the strains; resistance to imipenem and meropenem was 50 and 59% respectively, amikacin and gentamicin were both active against about 30% of the strains and colistin about 99%, with only one strain resistant. By comparison with tetracyclines, tigecycline and doxycycline showed a higher activity. In particular, tigecycline showed a MIC_90 _value of 2 mg/L and our strains displayed a unimodal distribution of susceptibility being indistinctly active against carbapenem-susceptible and resistant strains, these latter possessed OXA-type variant enzymes.

**Conclusion:**

In conclusion, tigecycline had a good activity against the MDR *A. baumannii *strains while maintaining the same MIC_90 _of 2 mg/L against the carbapenem-resistant strains.

## Background

*Acinetobacter baumannii *has emerged as one of the most problematic nosocomial pathogens to eradicate using available antimicrobial agents, and its occurrence has increased especially in patients admitted to intensive care units (ICUs). In a recent Italian survey, this microorganism ranked third among the most relevant pathogens in ICUs and was one of the most resistant microrganisms to all families of antibiotics [1]. The reasons for this emergence in nosocomial settings can be found among: i) its particular characteristics of being a colonizer of multiple body sites of hospitalized patients; ii) its ability to survive for a long time on inanimate surfaces; and iii) its tendency to be intrinsically resistant and to acquire new resistance determinants.

The clinical role of *A. baumannii *has been very controversial: it was considered a colonizer for a long time but recently, due to the report of severe illness with high associated mortality [2] and occurrence of some severe infections in bacteriemic patients, has been considered – by some clinicians – an indicator of severity, leading to the hypothesis that some changes can take place in this strain i.e. the acquisition of some virulence factors.

Treatment of *Acinetobacter *spp. infections has been limited to a few broad-spectrum agents including carbapenems, fluoroquinolones, ceftazidime, trimethoprim/sulfamethoxazole, sulbactam, amikacin and some tetracyclines. As resistance to carbapenems and other alternative drugs has emerged [3-7], often conferring a multi-drug-resistance (MDR) phenotype to this microrganism, the popularity of an old class of drugs, i.e. the polymyxin class of agents, has increased. However, colistin-resistant *A. baumannii *has been recently reported [8,9], and in spite of documented clinical success, colistin demonstrated dose-dependent nephrotoxicity [10].

In this context, it is mandatory to look at the activity of new antimicrobial agents and numerous reports have indicated that tigecycline, a novel compound belonging to a new class of antimicrobial agents – glycylcyclines -, displays inhibitory activity against *Acinetobacter *spp. [11-14]. This study sought to determine the *in vitro *activity of tigecycline and comparators against Italian carbapenem resistant and susceptible *A. baumannii *strains, isolated throughout Italy during a two-year surveillance study [1]. The presence of carbapenem-resistance genes was also investigated. These data can add new information to the *in vitro *activity of the most used antibiotics against this sample of epidemiologically representative Italian isolates.

## Methods

### Bacterial strains

One hundred and seven strains of *A. baumannii *were collected from 9 out of the 45 centres included in the 2002–2003 Italian surveillance study on microrganisms responsible for severe infections [1]. The first isolate of each patient was included in this study. These isolates were responsible for documented bloodstream and lower respiratory tract infections (82 strains), 15 strains were isolated from complicated Skin and Skin Structure Infections (cSSSI) and 3 strains from Intra-abdominal Infections (IAIs). The remaining 7 strains were isolated from urinary catheters. Species identification was centrally performed as previously described and reconfirmed by conventional methods using the API 20NE (Bio Merieux – SA- Marcy l'Etoile – France). Isolates were stored in Mueller-Hinton broth with 15% glycerol and frozen at -80°C prior to experiments.

### Antimicrobial agents and MIC determinations

MIC determinations were performed by broth microdilution in cation-adjusted Mueller-Hinton broth in accordance with CLSI guidelines [15]. MICs were interpreted with category designations according to CLSI criteria [15]. Breakpoints for tigecycline susceptibility were interpreted according to the FDA breakpoint of 2 mg/L.

Laboratory-grade standard reference powders of the following antimicrobials were obtained and reconstituted according to the manufacturers' instructions: tigecycline (Wyeth Research, PA, USA); amikacin, aztreonam, doxycycline, gentamicin, ciprofloxacin, cotrimoxazole, ceftazidime, tetracycline (Sigma Chemical Company, St. Louis, MO, USA), cefepime (Bruno Farmaceutici SpA Milan, Italy), ampicillin/sulbactam (Pfizer Italy), piperacillin/tazobactam (Wyeth Research, PA, USA), imipenem (Merck & Company, Rathway, NJ, USA), meropenem (Astra Zeneca, Waltham, MA, USA), and colistin (MP Biomedicals, Solon, Ohio, USA). Solutions of tigecycline were freshly prepared on the day of the experiments to avoid known degradation problems. All other antibiotics were freshly prepared as stock solutions and frozen at -20°C until their use.

### Time-kill studies

Five isolates (three imipenem-susceptible and two resistant strains) were chosen for time-kill studies: organisms were grown on Mueller-Hinton broth for 4 h (log phase of growth) and were further diluted in 20 ml of the same medium to yield a concentration of approximately 5 × 10^5 ^CFU/ml. Wells containing tigecycline at concentrations corresponding to the MIC and two and four times the MIC were tested for each strain. Aliquots (0.1 ml of broth) were removed from each well and serial dilutions were plated onto blood-agar plates after 0, 2, 4, 8 and 24 h of incubation. Colony counts were performed after 24 h of incubation at 36°C. Bactericidal activity was defined as a ≥3 log_10 _reduction compared with the initial inoculum [16].

### PCR of carbapenem resistance genes

PCR assays were carried out by using previously published primers and, in particular, for MBL-encoding genes *bla-*IMP, and *bla-*VIM we followed the protocol published by Pagani L. et al [17], and for amplification of genes encoding oxacillinases (*bla-*OXA_23_-like, *bla*-OXA_24_-like, *bla*-OXA_51_-like and *bla-*OXA_58_-like) the protocol published by Woodford N. et al [18] was used. The PCR assays were performed directly on colonies. PCR products were purified and sequenced (BMR Bio Molecular Research, Italy).

## Results

The 107 strains included in the study were collected from 9 centres in Italy, mainly located in the Central-South area of the country. Lungs and blood were the most common sources of the isolates (41.6% of all isolated came from lower respiratory tract infections, and 20.3% were isolated from blood cultures). In this survey, *A. baumannii *was also isolated from urinary catheters, complicated skin and skin structure (both 13.8%), and intra-abdominal infections (2.7%).

The results of the *in vitro *susceptibility testing, expressed as distribution, MIC_50_, MIC_90 _and percentage of resistance are presented in Table 1. The vast majority of strains of *A. baumannii *showed a multi-drug resistant phenotype (MDR), in particular, 53 strains were simultaneously resistant to three antibiotics, namely fluoroquinolones, ceftazidime and aminoglycosides or imipenem, while 44 strains were resistant to four antibiotics, the above three including carbapenems. 17% of strains were resistant also to ampicillin/sulbactam. More than 90% of strains were simultaneously resistant to ciprofloxacin, trimethoprim/sulphametoxazole, aztreonam, and ceftazidime; around 70% of strains were also resistant to gentamicin, amikacin, and tetracycline, while resistance to carbapenems was 50% for imipenem and 59% for meropenem. Among the tetracyclines, the unimodal distribution of susceptibility of tigecycline was comparable with that of doxycycline (93 and 94% susceptible, respectively). In our sample of strains colistin was active (106 out of 107 strains were susceptible). One strain was resistant to this drug, with a MIC of 16 mg/L.

**Table 1:** Distribution of MIC, MIC_50_, MIC_90 _(mg/l) for 107 isolates of *Acinetobacter baumannii *(MIC_50 _and MIC_90 _values are reported in BOLD cells and UNDERLINED numbers, respectively).

**MIC (mg/l)**																**% of**		
**Antibiotics**	0.015	0.03	0.06	0.12	0.25	0.5	1	2	4	8	16	32	64	128	256	**S**	**I**	**R**
**Tigecycline**					3	15	26	56	3	4						93	3	4
**Amikacin**									27	4	2	16	58			30	0	70
**Gentamicin**								8	18	45	6	30				24	0	76
**Ciprofloxacin**						5	1		1	100						5	0	95
**Trimethoprim-sulfamethoxazole**							5		3	11	88					4	0	96
**Aztreonam**									3	1	1	3	99			4	1	95
**Cefepime**									8	29	48	13	9			34	44	22
**Ceftazidime**									4				103			4	0	96
**Piperacillin-tazobactam**											55	4	1	2	45	51	5	44
**Imipenem**								26	9	18	11	39	2		2	33	17	50
**Meropenem**							1	5	5	33	7	18	27	11		10	31	59
**Doxycycline**			1	1	3	5	7	81	3	1	3	2				94	1	5
**Tetracycline**						1	6	7	2	12	13	9	19	31	7	15	12	73
**Colistin**	1	1	1	14	19	20	30	20			1					99	0	1
**Ampicillin/sulbactam**						1	3	22	19	31	13	7	5	4	2	71	12	17

The molecular characterization of the carbapenem-resistance gene content of the 58 isolates that were resistant to meropenem is shown in Table 2. The *bla*_OXA51 _and *bla*_OXA58_-like, were the most diffused genes: 43 strains carried both these determinants and 14 strains carried the *bla*_OXA51_-like gene. In our sample of carbapenem-resistant strains, only one carried the *bla*_oxa23_-like together with the *bla*_oxa51 _gene; all strains were negative for MBL (*bla*_VIM _and *bla*_IMP_) enzymes (table 2). Six out of the 14 strains carrying the *bla*_OXA-51_-like gene, demonstrated a susceptible or intermediate level of resistance to imipenem while they were resistant to meropenem, suggesting that other mechanisms can be responsible for carbapenem resistance in these strains. In the 43 strains carrying the *bla*_OXA51 _and *bla*_OXA58_-like genes there is an association between the presence of the OXA-58 enzyme and resistance to carbapenems.

**Table 2:** Tigecycline in carbapenem-resistant isolates carrying different OXA-like genes

**Bla_OXA _genes**	**N. of strains**	**Imipenem mg/L**			**Meropenem mg/L**			**Tigecycline mg/L**	
		≤4	8	≥16	≤4	8	≥16	≤2	**≥ 4**
OXA-23/OXA-51	1	0	0	1	0	1	0	1	0
OXA-51	14	3	3	8	0	0	14	12	2
OXA-51/OXA-58	43	2	2	39	0	0	43	41	2

A preliminary observation on the level of resistance to carbapenems demonstrated that meropenem is a more hydrolysable substrate with respect to imipenem, and in all cases, tigecycline remained active in all carbapenem-resistance-strains.

Time-kill studies with increasing concentrations of tigecycline above the MIC, demonstrated that this antibiotic has a maximal killing effect near the MIC, without any increased rate at multiples of the MIC (Table 3). Its bacteriostatic activity was similar in all strains tested irrespective of their resistance to other antibiotics such as carbapenems.

**Table 3:** Time kill results, expressed as average reduction of log_10 _of CFU/ml, of tigecycline against five *A. baumannii *strains with different susceptibilities to carbapenems.

**Concentration of tigecycline**	**2 h**	**4 h**	**8 h**	**24**
MIC	0.6 × 10^-1^	3 × 10^-2^	3 × 10^-2^	3 × 10^-2^
2 × MIC	0.8 × 10^-1^	2.4 × 10^-2^	1.3 × 10^-2^	1.4 × 10^-2^
4 × MIC	1 × 10^-1^	1.4 × 10^-2^	1.2 × 10^-2^	1.2 × 10^-2^

## Discussion

*Acinetobacter baumannii *is one of the most important nosocomial pathogens, able to cause severe infections, occurring principally in immunosuppressed patients, in patients with serious underlying diseases, or subjected to invasive procedures and treatment with broad-spectrum antibiotics [19]. This microorganism is also able to spread in high-risk wards, causing outbreaks. Nevertheless, until recently, carbapenems represented a viable choice for treating infections caused by this organism due to a diffused sensitivity to both imipenem and meropenem. In Italy, until 2000, very few strains were resistant [20], and only few genotypes appeared to occur in multiple hospitals causing outbreaks [21-26]. The recent Italian survey previously cited [1] demonstrated that *A. baumannii *was the third pathogen for severity in high-risk wards, frequently derived from nosocomial pneumoniae (VAP) or bacteremia. In our experience, nosocomial isolates of this microorganism were also co-pathogens in cSSSI and a few strains also came from IAIs. Approximately half of the 107 single isolates were MDR to three or four classes of antibiotics, including carbapenems, while they were almost fully susceptible to tigecycline, doxycycline and colistin. In our results, tigecycline is active, with a MIC_90 _of 2 mg/L against 93% of strains with maximum killing at the MIC concentrations.

The activity of tigecycline in our Italian isolates is comparable with that of many other national and global studies [12,27-29], including the worldwide program T.E.S.T.(Tigecycline Evaluation and Surveillance Trial, [12] that reported a MIC_90 _value of 1 mg/L for a large number of *A. baumannii *isolates (4353) worldwide. The same study reported the isolation of 14.8% imipenem-resistant strains. Our data are also in line with the results obtained in other European studies [30,31] in which tigecycline demonstrated its activity with MIC_90 _of 2 mg/L against strains showing a multiple resistance phenotype.

*A. baumannii *isolates resistant to various classes of antibiotics are emerging worldwide, and the recent resistance or reduced susceptibility to carbapenems, is considered a serious clinical problem due to the role of first choice therapy that these drugs have had until now.

Numerous mechanisms, including decreased permeability, efflux pump overexpression, and carbapenemase production, can be responsible for the resistance to carbapenems [7,32,33]. Class B (IMP and VIM enzymes) and D (oxacillinases) beta-lactamases are the most important group of enzymes able to hydrolyze carbapenems: although MBLs are not the predominant enzymes in *A. baumannii*, several IMP-enzymes have been described in various countries [34-37] while VIM-1, first identified in Italy in a strain of *P. aeruginosa *[38], is now reported in Korea [39], in Poland [40] and, as sporadic isolates, in many other parts of the world. Most worrisome are the carbapenemase-hydrolyzing oxacillinase (OXA) clusters that have been identified in *A. baumannii *in nine major subgroups [7,41,42], including the most diffused throughout the world OXA-23, OXA-24, OXA-51, OXA-58 enzymes and their variants [43]. The significant contribution of these enzymes to carbapenem resistance in *A. baumannii *has been emphasized, particularly when they are accompanied by IS_Aba1 _and IS_Aba3 _in the naturally occurring plasmid [44], due to the fact that these sequences can provide the promoter required for expression of linked antibiotic-resistance genes. The OXA-51/69-like beta-lactamases are, instead, "naturally occurring" chromosomal enzymes in this species, isolated from four continents, and their expression varies according to the presence of IS_Aba1 _as previously discussed [45]. All these enzymes have been found in different isolates from various countries [24,46-49]. In our strains, OXA-51 alone or associated with other enzymes was found. As recently suggested [32], these enzymes are very poor carbapenemases and may be natural chromosomally encoded in the vast majority of *A. baumannii *isolates, regardless of their susceptibility or resistance to carbapenems. OXA-58 enzymes are the most diffused oxacillinases in 43 strains of *A. baumannii*. As recently demonstrated, these enzymes may contribute to carbapenem resistance [32,45], although it cannot be excluded that other mechanisms may additionally affect the activity of these drugs.

The recent emergence of high-resistance rates to tigecycline in multiple clones of MDR strains is also cause for concern. Various Authors [50-52] have reported a few cases of high tigecycline-resistance in nosocomial isolates: the involvement of an overexpression of the AdeABC multidrug efflux pump in the decreased susceptibility of tigecycline in these strains was postulated [52,53]. To date, tigecycline has been approved for the treatment of complicated intra-abdominal and complicated skin and skin structure infections; the well documented activity of this drug against *A. baumannii *isolates and the high concentration reached by this drug in alveolar cells (77.5 fold higher than serum)[29], have indicated its use in infections sustained by these MDR microorganisms [54], despite the lack of approved clinical indications and criteria for *in vitro *susceptibility testing. In this situation, due to the importance that tigecycline can have in the treatment of MDR *A. baumannii *infections, it is absolutely mandatory to monitor isolates for evidence of acquired microbiological resistance, requiring further investigations to understand better all the possible mechanisms underlying this phenomenon.

## Conclusion

Our results have shown that the MDR resistance phenotype is very diffused in *A. baumannii *isolated from severe infections in Italy. We have documented that almost half of the strains are also resistant to carbapenems and, as published by other Authors [55], also colistin has not escaped development of resistance: in fact, we isolated one strain resistant to this drug. Carbapenem-resistance in these strains is due to the presence of three different oxacillinases and the most diffused hydrolytic enzymes are the OXA-51/OXA-58 enzymes, carried in 43 strains. Tigecycline shows a potent antibacterial activity with a unimodal distribution of MIC and a MIC_90 _of 2 mg/L; the drug has indeed a bacteriostatic effect at the MIC concentration, and its activity is not influenced by the other mechanisms of resistance carried in these microorgansims, including the resistance to carbapenems. Furthermore recent studies demonstrated that tigecycline achieved maximal killing near the MIC in this species and concentration escalation studies demonstrated that this drug needs to approach concentrations higher than those currently achieved in the bloodstream to adequately treat *A. baumannii *infections [13].

These *in vitro *results, obtained in a representative sample of Italian isolates – all from documented severe infections – need to be confirmed by the clinical efficacy of this drug from Phase 3 clinical trials regarding the treatment of nosocomial pneumonia and other infections sustained by this microrganism. For the moment, the evaluation of the microbiological and pharmacological profile of tigecycline in each patient and the careful assessment of susceptibility can assist physicians in deciding on tigecycline use.

## Competing interests

GN and SS received funding from Wyeth – Italy.

## Authors' contributions

MML participated in the study design, interpretation of the results, and co-drafted the manuscript; TG participated in the study design, co-performing MIC and killing curves, analysis and interpretation of data; GF participated in the study design, co-performing DNA extractions and PCR experiments; NMV and ND participated in design and provided the strains for the study; CA, FG and GN participated in design, in the coordination of the study and in the revision of the manuscript; SS participated in the design the study, interpretation of the data, co-drafted the manuscript and participated in the final revision
